# Alterations in gene expressions of Caco-2 cell responses to LPS and ploy(I:C) stimulation

**DOI:** 10.7717/peerj.15459

**Published:** 2023-06-07

**Authors:** Ge Qin, Yuanjie Zhao, Yating Gan, Xiaomei Yu, Yifan Zhao, Hui Peng, Shaoming Fang

**Affiliations:** 1 Fujian Agriculture and Forestry University, Fuzhou, China; 2 Hainan University, Haikou, China

**Keywords:** LPS, ploy(I:C), Caco-2, Intestinal epithelium barrier, Transcriptome sequencing, qRT-PCR

## Abstract

The intestinal epithelium barrier serves as a highly dynamic immunologic frontier in the defense against invading pathogenic bacteria and viruses. Hence, understanding of the complicated underlying relationship between enteric pathogens and the intestinal epithelium barrier is vital for developing strategies to improve the intestinal health of farm animals. To this end, Caco-2 cells were stimulated by 1 µg/ml lipopolysaccharide (LPS) for 24 h and 5 µg/ml polyinosinic-polycytidylic acid (ploy(I:C)) for 4 h to imitate bacterial and viral infection processes, respectively. The specific alterations in gene expression of Caco-2 cells after stimulation were characterized by transcriptome sequencing. Seventy differentially expressed genes (DEGs) were identified under LPS exposure, and 17 DEGs were observed under ploy(I:C) exposure. We found that most DEGs were specific, and only one common DEG *SPAG7* was observed. Gene Ontology (GO) annotation analysis indicated that all DEGs identified in the different treatments were mainly derived from GO terms related to the maintenance of cellular homeostasis. Moreover, specific DEGs such as *SLC39A10*, *MT2A*, and *MT1E* regulated by LPS treatment, while *IFIT2* and *RUNX2* mediated by ploy(I:C) treatment, which are derived from immune function modulation related GO terms, were confirmed by both transcriptome sequencing and qRT-PCR. In addition, both transcriptome sequencing and qRT-PCR results verified that LPS specifically down-regulated the DEGs *INHBE* and *ARF6*, which are involved in inflammation responses related to the Kyoto Encyclopedia of Genes and Genomes (KEGG) pathway including the TGF-beta signaling pathways and the Ras signaling pathway. Ploy(I:C) uniquely suppressed the DEGs *GABARAP* and *LAMTOR3*, which participated in viral replication-associated pathways including autophagy and mTOR signaling pathway.

## Introduction

Since the ban on growth promoting antibiotics in animal feed, health dysbiosis problems have become a major issue, particularly in intensively reared farm animals (Mingmongkolchai & Panbangred, 2018). Intestinal health is vital for the general health of farm animals due to the key roles of intestine in the nutrient digestion and absorption, maintenance of microbiome homeostasis, mucosal barrier function, and mucosal immune responses (Ghiselli et al., 2021). The intestinal epithelium barrier is one of the main components of the intestine, and it functions as the central line of defense against both commensal microorganisms and invading enteric pathogens. Dysfunction of the intestinal epithelial barrier can lead to great susceptibility to infectious diseases and increased mortality rate in farm animals (Ducatelle et al., 2018).

Intestinal epithelial cells are essential for barrier development and function modulation but are constantly challenged by pathogenic bacteria and viruses. Lipopolysaccharide (LPS) is regarded as a potent immunogenic component of Gram-negative pathogenic bacteria and is well known as a common immune stressor of intestinal epithelial cells (Wu et al., 2020). LPS exposure can stimulate localized or systemic inflammation and lead to intestinal dysfunction in farm animals. For example, Xu et al. (2021) reported that piglets that received an intraperitoneal injection of LPS exhibited increased pro-inflammatory cytokines levels and decreased tight junction protein expression, villus height, and the villus height/crypt depth ratio, which resulted in an inflammatory response and the altered ileum morphology, respectively. Wu et al. (2021) reported that LPS administration in chickens is capable to inducing inflammatory responses *via* down-regulating the synthesis of immunoglobulins and causing intestinal epithelium injuries by damaging the villi structure and mucosal layer. Sullivan et al. (2022) demonstrated that LPS exposure triggered a systemic inflammatory response in dairy cattle, as evidenced by marked leukopenia and thrombocytopenia and increased level of cortisol.

Polyinosinic-polycytidylic acid (ploy(I:C)) is a mimic of viral double-stranded RNA that is widely used to establish viral infection models (Bao, Hofsink & Plosch, 2022). The ploy(I:C) transfection in intestinal epithelial cells results in the expression of an extensive collection of innate immune response genes and proteins. For instance, the porcine intestinal epithelial cells treated with ploy(I:C) exhibited significantly increased chemokine and monocyte chemoattractant protein expression (Mizuno et al., 2020). Ploy(I:C) treatment activates antiviral defense genes such as interferon (IFN)-*α*, IFN- *β*, and Toll-like receptor 3 (TLR3), which are involved in the innate antiviral immune response in bovine intestinal epithelial cells (Albarracin et al., 2019).

In this study, Caco-2 cells were used as a model of the intestinal epithelial barrier, and they were stimulated by LPS and ploy(I:C) to mimic pathogenic bacterial and viral infection processes, respectively. The specific alterations in gene expression were investigated by transcriptome sequencing and confirmed *via* qRT-PCR. Our findings will provide basic knowledge for understanding the complex relationships between invading pathogenic microorganisms and the intestinal epithelial barrier, which is important for developing strategies to improve the intestinal health of farm animals.

## Materials & Methods

### Caco-2 cell culture and treatment

Caco-2 cells were obtained from the American Type Culture Collection (Manassas, VA, USA). Caco-2 cells were cultured in Dulbecco’s Modified Eagle’s Medium (DMEM, HyClone, Logan, USA) supplemented with 10% fetal bovine serum (FBS, HyClone): 100 U penicillin (Beyotime, Jiangsu, China) and 0.1 mg/ml streptomycin (Invitrogen, Waltham, MA, USA). The cells were seeded in six cm transwell chambers (Corning, Corning, NY, USA) at a density of 2.5 × 10^5^ cells per milliliter of complete culture solution. The cells were incubated in an atmosphere of 5% CO_2_ and 37 °C incubator. When the cells had grown to 80%–90% confluence, the spent cell culture media from the six cm transwell chambers was removed and discarded, and the cells were washed with 2 ml of phosphate buffered saline (PBS, HyClone, Logan, UT, USA). The culture medium was changed every 2 days to maintain the cells. Monolayered cells were collected by adding 0.25% trypsin (Procell, Wuhan, China) for 3 min, and then they were seeded at 2.5 × 10^5^ cells/well in 5.0 ml complete culture solution for 24 h. In the LPS exposure experiment, the Caco-2 monolayers were treated with 1 µg/ml LPS (Sigma-Aldrich, St. Louis, MO, USA) for 24 h (Huang et al., 2003). In the poly(I:C) exposure experiment, the Caco-2 monolayers were transfected with poly(I:C) (Invitrogen, Waltham, MA, USA) at a final concentration of 5 µg/ml complexed to 10 µg/ml Lipofectamine 3000 (Invitrogen, Waltham, MA, USA) for 4 h (Mendoza et al., 2012). All cell stimulations were performed on the separate occasions, using three independent biological samples per treatment.

### Total RNA extraction and transcriptome sequencing

Cells were gently washed with sterile PBS three times, and total RNA extraction was performed by using NucleoZOL reagent (Gene Company, Hong Kong, China) according to the manufacturer’s instructions. The RNA integrity and concentration were determined by 1% agarose gel and NanoDrop 2000 Spectrophotometer (Thermo Scientific, Waltham, MA, USA), respectively. RNA quality was assessed by an Agilent 2100 Bioanalyzer (Agilent Technologies Inc., Santa Clara, CA, USA). The qualified RNA samples were sent to Biomarker Technologies Corporation (Beijing, China) for transcriptome sequencing analysis. Briefly, 1 µg of qualified RNA per sample was used for cDNA synthesis. The cDNA library was prepared by using the NEB Next Ultra II RNA Library Prep Kit for Illumina (NEB, Ipswich, MA, USA) according to the manufacturer’s recommendations. The constructed library was sequenced on an Illumina HiSeq 2500 platform (Illumina Inc., San Diego, CA, USA) for 2 × 150 bp reads. Raw data were transformed into clean data by removing reads containing adapters, reads containing poly-N and low-quality reads *via* the in-house Perl scripts. Then, the Q30, GC content, and sequence duplication level of the clean data were calculated.

### Transcriptome alignment and functional analysis

The clean reads were aligned to the reference genome *Homo sapiens* Hg38 (GRCh38, https://ftp.ncbi.nlm.nih.gov/genomes/refseq/vertebrate_mammalian/Homo_sapiens/reference/) using Hisat2 (v2.0.4) (Kim, Langmead & Salzberg, 2015). Transcriptome assembly was performed using StringTie (v1.3.1) (Pertea et al., 2015). Gene expression levels were quantified with fragments per kilobase per million mapped reads (FPKM). Principal component analysis (PCA) was performed using gene expression data to show the differences in the gene expression profile of each group. DESeq2 (v1.10.1) (Love, Huber & Anders, 2014) was then used to identify differentially expressed genes (DEGs) between the treated and control samples with the criteria of | log2 fold change |≥ 1 and FDR adjusted *P* < 0.05. To obtain Gene Ontology (GO) annotations for the DEGs, PANTHER17.0 (http://www.pantherdb.org/) was used to retrieve biological process, molecular function, and cellular component terms with the following steps: upload the gene ID list, choose *Homo sapiens*, and view the functional classification as a gene list (Li et al., 2019). WebGestalt (http://www.webgestalt.org/) was applied to uncover statistical enrichment of the DEGs in the Kyoto Encyclopedia of Genes and Genomes (KEGG) pathways using the following parameters: minimum number of genes for a category ≥1 and FDR adjusted *P* < 0.05 (Liao et al., 2019).

### Validation of DEGs by quantitative real-time PCR (qRT-PCR)

To confirm the results of transcriptome sequencing, six specific DEGs related to LPS exposure responses and six DEGs specifically related to responses to poly(I:C) treatment were chosen for verification by qRT-PCR. The primer sequences and related information are shown in Table 1. PrimeScript RT Reagent Kit (TaKaRa, Shiga, Japan) was used to synthesize cDNA from 1 µg of total RNA for each sample. The qPCR analysis was performed using the ABI 7500 Real-Time PCR System (Thermo Fisher) with Power SYBR Green PCR Master Mix (Thermo Fisher). The reaction mixture comprised 1.5 µL diluted cDNA, 0.5 µL of each forward and reverse primer, 3 µL sterile deionized water, and 4.5 µL Master Mix. The PCR conditions were as follows: 95 °C for 10 min, 40 cycles of 95 °C for 15 s, 60 °C for 30 s, 72 °C for 30 s. Three RNA samples from each group were run in triplicate. The relative expression level of each gene was normalized to the endogenous control gene GAPDH, and expression ratios were calculated using the 2^−ΔΔCt^ method.

### Statistical analysis

All data were analyzed using Student’s *t*-test in R software (v4.0.3). False discovery rate (FDR) adjusted *P* < 0.05 was accepted as significant. The “**” represents for *P* < 0.01, “***” for *P* < 0.005, and “****” for *P* < 0.001. The Venn plot was produced by VennDiagram R package and the other plots were visualized by ggplot2 R package.

## Results

### Transcriptome sequencing and alignment quality assessment

As shown in Table 2, after removing low quality reads and adapters, a total of 203.88 million and 183.47 million clean reads were obtained from the LPS and poly(I:C) exposure experiments. An average of 36.08 million, 31.87 million, 31.77 million, and 29.38 million clean reads were obtained from cDNA libraries of the LPS exposure group, LPS control group, poly(I:C) exposure group, and poly(I:C) control group, respectively. Quality assessment of the sequencing data showed that the Q30 value of each group was over 94%. The GC and AT contents of each group were almost equal. Then, clean reads were aligned to the reference genome GRch38 by HISAT2. A total of 192.48 million and 173.90 million clean reads from LPS and poly(I:C) exposure experiments were successfully mapped, with 185.79 million and 168.28 million were uniquely mapped. Both the mapping efficiency and uniquely mapped efficiency of each group were more than 90%. These results indicated that the sequencing data were high quality and reliable, and could be used for subsequent analysis.

**Table 1:** Primers used for qRT-PCR validation of differentially expressed genes.

Gene name	Forward primer (5′–3′)	Reverse primer (5′–3′)	Tm (°C)
INHBE	ACTACAGCCAGGGAGTGTGG	AGTGAGCAGGGAGCTGTAGG	FP:59.19 RP:59.23
MT2A	AGCTTTTCTTGCAGGAGGTG	GCAACCTGTCCCGACTCTA	FP:59.62 RP:59.07
MT1E	CTCATTGCCCGTGTCATTC	AGAACCCAGACCCAGAGGA	FP:60.04 RP:60.09
SLC39A10	TTTCACTCACATAACCACCAGC	GTGATGACGTAGGCGGTGATT	FP:59.58 RP:59.43
APOBEC3	TTGGAAGGCATAAGACCTACCTG	CAGAGAAGATTCTTAGCCTGGTTGTG	FP:60.90 RP:60.83
ARF6	ATGGGGAAGGTGCTATCCAAAATC	GCAGTCCACTACGAAGATGAGACC	FP:61.30 RP:61.45
IFIT2	AGCGAAGGTGTGCTTTGAGA	GAGGGTCAATGGCGTTCTGA	FP:59.50 RP:59.90
RUNX2	CGCCTCACAAACAACCACAG	TCACTGTGCTGAAGAGGCTG	FP:60.03 RP:60.02
GABARAP	GGGTGCCGGTGATAGTAGAA	AATTCGCTTCCGGATCAAG	FP:59.96 RP:59.96
PLXNC1	AACTGTTCCCTTCCTTGACTAC	TCGTTGGCGTCTCTGTTATG	FP:60.03 RP:60.10
LAMTOR3	CTGAAGTGACAGCGGAGAGA	TCGCAGGATCAATCTCCAC	FP:60.09 RP:60.23
SPAG7	CCGCCTGAAGAAACTACAAG	ATCATGTAGTATGCTCCTCTC	FP:59.89 RP:59.63
GAPDH	GGTGTGAACCATGAGAAGTATGA	GAGTCCTTCCACGATACCAAAG	FP:60.41 RP:60.68

**Notes.**

FP forward primer RP reverse primer

**Table 2:** Sequences quality and reads mapping of different samples.

Samples	Clean reads	Q30%	GC%	Mapped reads	Unique mapped reads
LPS1	38,126,744	94.29%	50.65%	36,008,258 (94.44%)	34,901,177 (91.54%)
LPS2	35,203,444	93.77%	50.41%	33,152,407 (94.17%)	31,547,155 (89.61%)
LPS3	34,923,253	94.05%	49.92%	32,860,710 (94.09%)	31,834,282 (91.15%)
CLPS1	30,683,298	94.05%	49.73%	28,896,011 (94.18%)	27,837,621 (90.73%)
CLPS2	33,420,602	94.43%	50.11%	31,682,117 (94.80%)	30,692,212 (91.84%)
CLPS3	31,530,842	94.47%	50.23%	29,882,067 (94.77%)	28,985,826 (91.93%)
PolyIC1	32,413,772	94.56%	50.62%	30,796,719 (95.01%)	29,890,039 (92.21%)
PolyIC2	26,512,197	94.6%	51.07%	24,992,758 (94.27%)	24,153,693 (91.10%)
PolyIC3	36,393,528	94.18%	50.92%	34,436,214 (94.62%)	33,286,709 (91.46%)
CPolyIC1	30,683,298	94.05%	49.73%	29,116,185 (94.86%)	28,272,727 (92.12%)
CPolyIC2	33,420,602	94.43%	50.11%	27,466,988 (94.85%)	26,540,968 (91.65%)
CPolyIC3	31,530,842	94.47%	50.23%	27,094,064 (95.05%)	26,144,517 (91.71%)

**Notes.**

LPS1-3 LPS treatment group CLPS1-3 Control group of LPS treatment PolyIC1-3 ploy(I:C) treatment group CPolyIC1-3 Control group of ploy(I:C) treatment

### Differentially expressed genes (DEGs) in Caco-2 cells in response to LPS and poly(I:C) simulation

**Figure 1:**
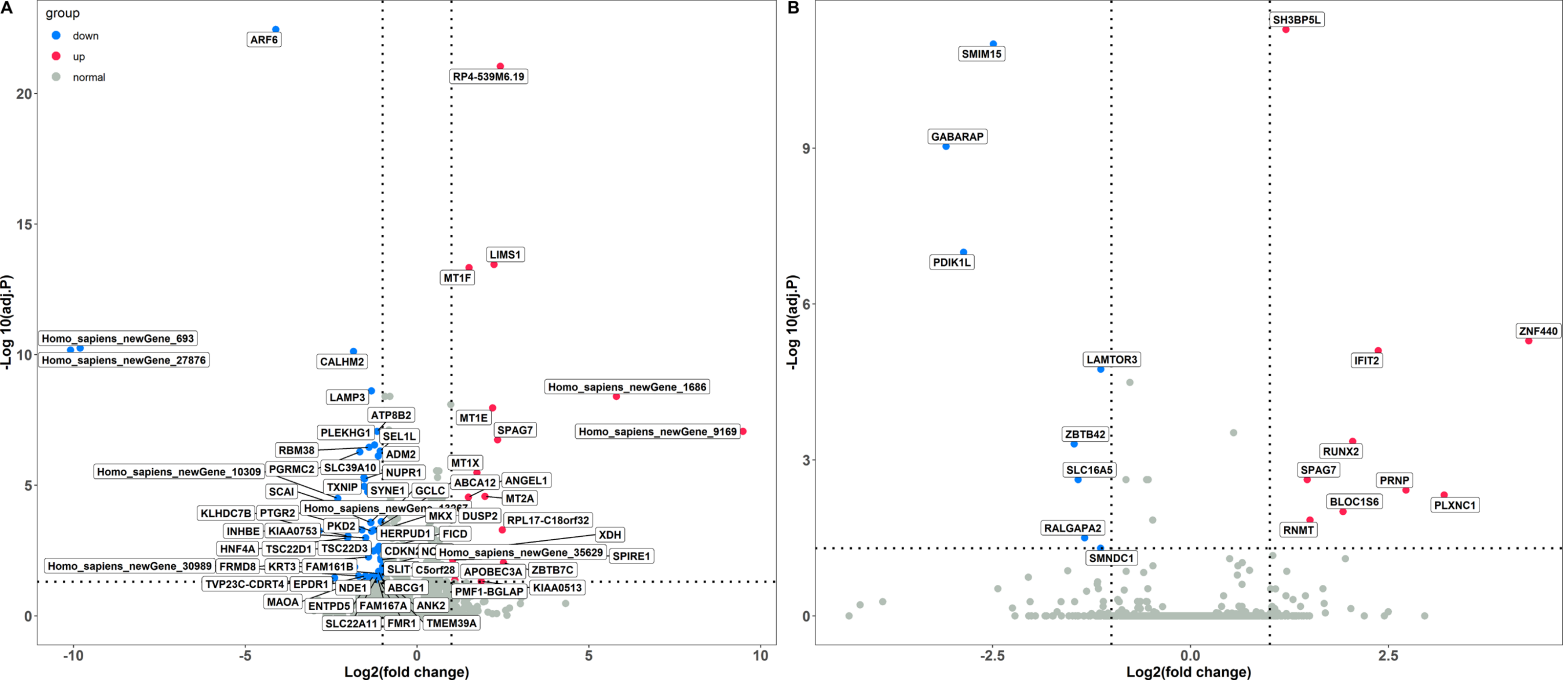
Identification of DGEs in different treatment groups. (A) DGEs between the LPS treatment and control groups. (B) DGEs between the poly(I: C) treatment and control groups.

The PCA analysis showed that the Caco-2 cells in different treatment groups formed distinct clusters, which implied potential differences in the gene expression profile in each group (Fig. S1). Subsequently, DESeq2 was used to identify the DEGs with |log2 fold change |≥ 1 and FDR adjusted *P* < 0.05. Exposure to LPS resulted in a total of 70 differentially expressed genes, corresponding to 18 up-regulated and 52 down-regulated genes, respectively (Fig. 1A and Table S1). In total, 17 DEGs were identified between the poly(I:C) simulation and control groups, among them, nine were up-regulated, while eight were down-regulated (Fig. 1B and Table S2). When we compared the DEGs from LPS and poly(I:C) simulation, most of them were specific to the corresponding treatment, and only the up-regulated gene *SPAG7* (sperm associated antigen 7) was shared (Fig. S2).

### Functional annotation and classification of the DEGs

**Figure 2:**
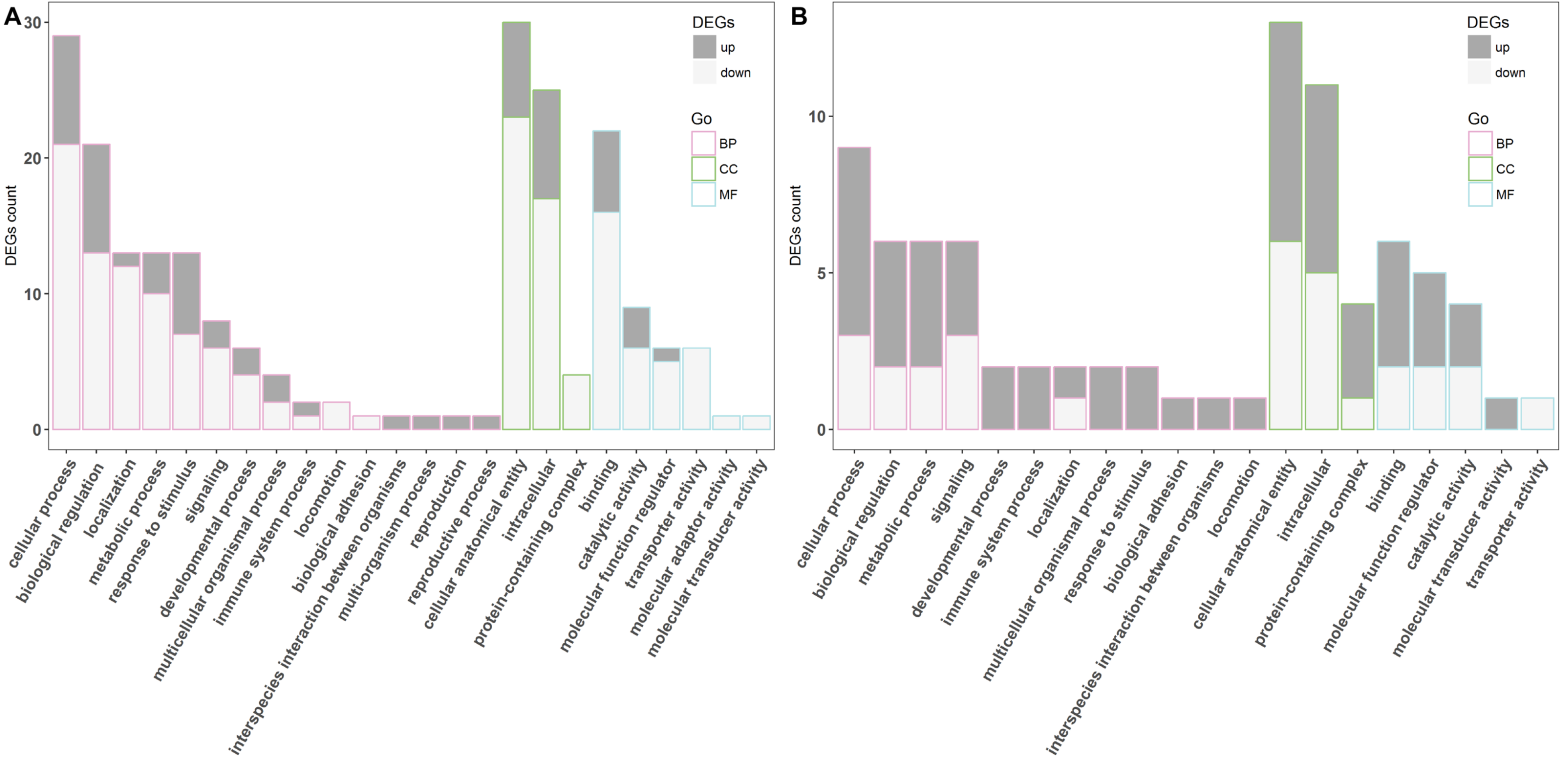
The GO classification of DGEs. The LPS exposure group (A) and poly(I: C) exposure group (B). BP, CC, MF represents for biological process, cellular component, and molecular function, respectively.

To investigate the biological importance of the differentially expressed genes, gene ontology (GO) functional enrichment analysis was performed. In the LPS-treated group, 70 DEGs were classified into the following three functional categories: biological process, cellular component, and molecular function, composed of 15, three, and six subcategories, respectively (Fig. 2A). In the biological process category, cellular process, biological regulation, localization, metabolic process, and response to stimulus were the dominant functional terms. Cellular anatomical entity, intracellular, and protein-containing complex were the top functional terms in the cellular component category. Most of the DEGs in the category of molecular function were related to binding, catalytic activity, molecular function regulator, and transporter activity. Meanwhile, all DEGs in the poly(I:C)-treated group were also classified into the same major functional categories, but with 12, three, and five subcategories, respectively (Fig. 2B). Cellular process, biological regulation, metabolic process, signaling, and immune system process were the most represented functional terms within the biological process category, while binding, molecular function regulator, and catalytic activity were the top three functional terms within molecular function. In addition, the DEGs belonging to the cellular component category were further fell into the same functional terms as mentioned above in the LPS exposure treatment.

**Figure 3:**
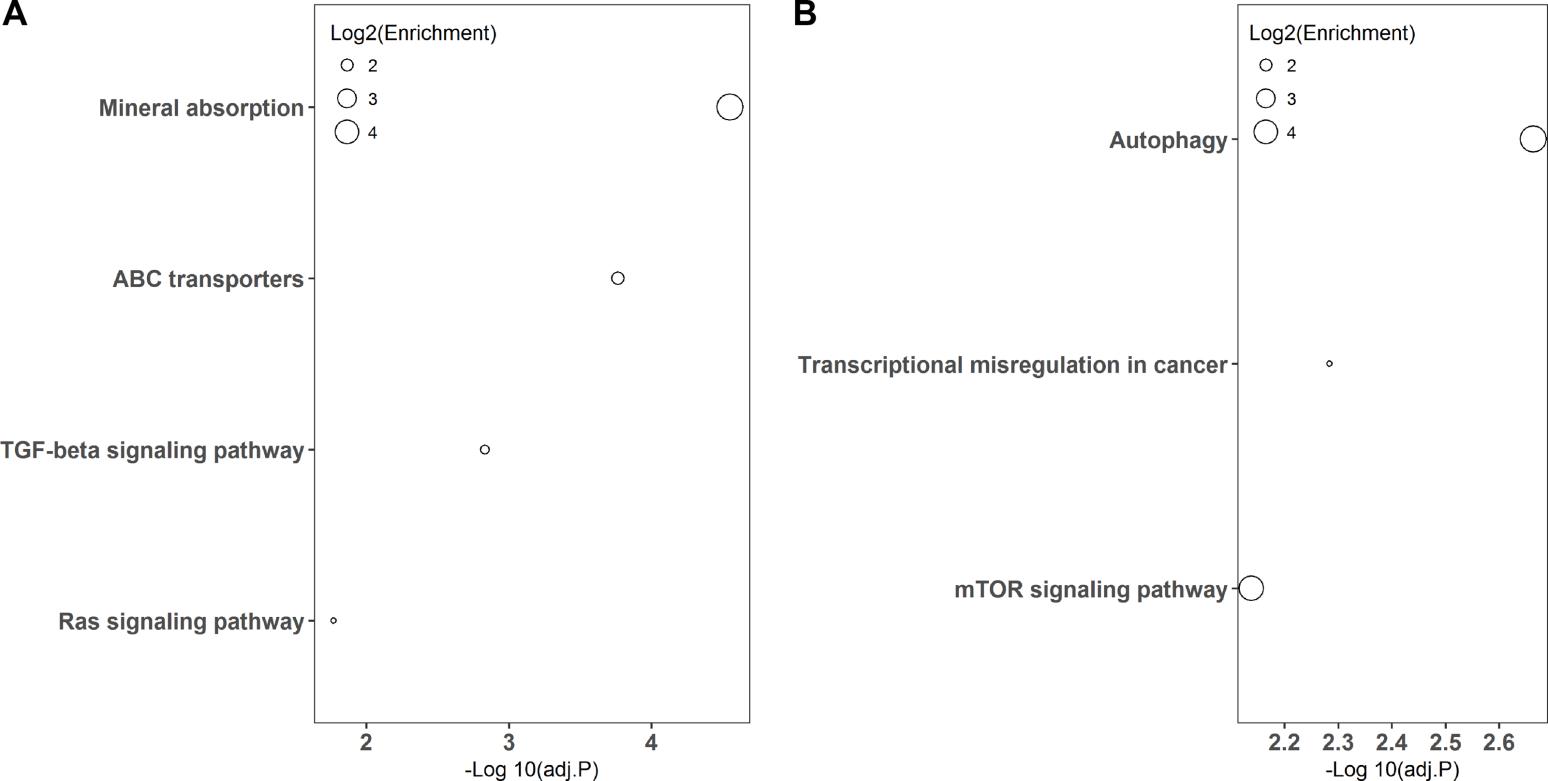
KEGG pathway enrichment analysis of DGEs. LPS simulation group (A) and poly(I: C) simulation group (B).

KEGG pathway-based overrepresentation analysis was used to uncover the biological pathways related to the DEGs. In total, DEGs of LPS and poly(I:C) simulation were assigned to four and three KEGG pathways, respectively (Fig. 3). Among these, the TGF-beta signaling pathway and Ras signaling pathway are associated with inflammatory responses, and autophagy and mTOR signaling pathway are related to antiviral responses.

### Validation of the transcriptomic sequencing results by qRT-PCR

**Figure 4:**
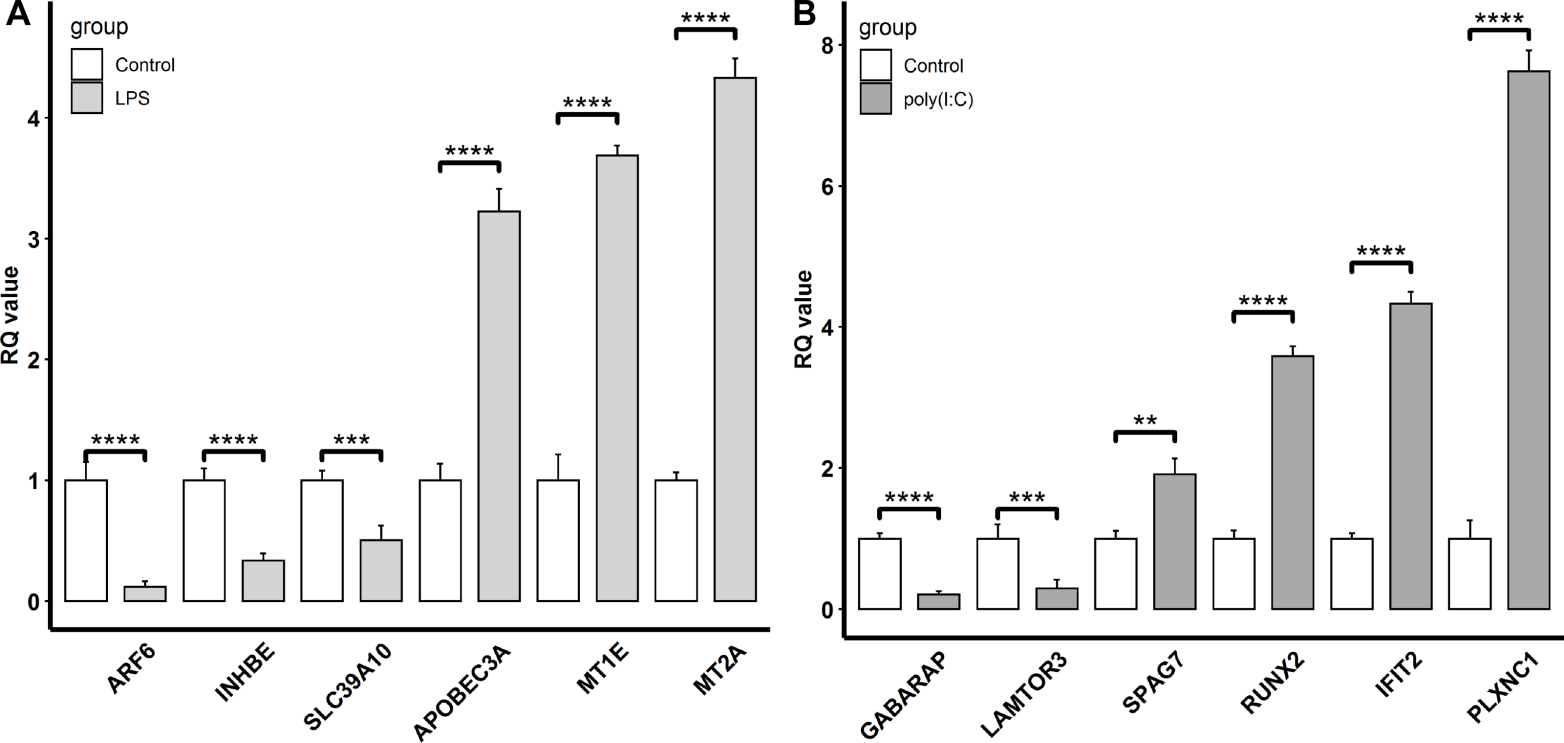
Verification of the selected DEGs identified. LPS stimulation group (A) and poly(I: C) stimulation group (B) by qRT-PCR. “**”, *P* < 0.01, “***”, *P* < 0.005, “****”, *P* < 0.001.

To confirm the reliability of the transcriptomic sequencing data, *INHBE*, *MT2A*, *MT1E*, *SLC39A10*, *APOBEC3A*, and *ARF6* regulated by LPS exposure, and *IFIT2*, *RUNX2*, *GABARAP*, *PLXNC1*, *LAMTOR3*, and *SPAG7* modulated by ploy(I:C) treatment were selected for verification by qRT-PCR. Compared to transcriptome sequencing analysis, the similar expression patterns of these representative genes were observed by qRT-PCR (Fig. 4). In the LPS exposure group, the expression of *APOBEC3A*, *MT1E*, and *MT2A* was significantly up-regulated (*P* < 0.005), while *ARF6*, *INHBE*, and *SLC39A10* exhibited markedly decreased expression levels (*P* < 0.005). In the ploy(I:C) treatment group, the expression of *SPAG7*, *RUNX2*, *IFIT2*, and *PLXNC1* was enhanced (*P* < 0.01) , but that of *GABARAP* and *LAMTOR3* was suppressed (*P* < 0.01).

## Discussion

Livestock commonly experience a number of stresses in their lifetime such as weaning, transportation, overproduction, and diseases. Diseases are considered as the most significant stressors in animal production systems, which exert deleterious effects on animal production performance. Intestinal homeostasis plays a crucial role in maintaining general health. However, the intestinal equilibrium is frequently disturbed by bacterial and viral invasions, which results in various diseases. Hence, understanding the alterations in intestinal epithelial cells during the processes of bacterial and viral infections is important for the prevention and treatment of diseases. Caco-2 cells are accepted as an *in vitro* model of the intestinal epithelium (Graber et al., 2018) were used to gain insights into the molecular mechanisms of the specific responses of intestinal epithelium cells to LPS (bacterial) and poly(I:C) (viral) stimulations by transcriptome sequencing in the present study.

A total of 70 DEGs were detected in Caco-2 cells stimulated by LPS, among which 12 were up-regulated and 58 were down-regulated (Fig. 1A). To investigate the biological functions enriched by the DEGs, GO annotation was used for gene clustering (Fig. 2A). The top 5 GO terms among the three functional categories were cellular anatomical entity, cellular process, intracellular, biological regulation, and binding. These results are similar to those from porcine intestinal epithelial cell (IPEC-J2) exposure to LPS (Dong et al., 2019), which suggests that LPS significantly affects intestinal cellular homeostasis.

Meanwhile, DEGs involved in the response to the LPS simulation derived from the GO terms such as immune system process and response to stimulus were also observed. Among these DEGs, the up-regulated *APOBEC3A* (apolipoprotein B mRNA editing catalytic polypeptide-like) and down-regulated *SLC39A10* (solute carrier family 39 member 10) were commonly found and validated by qRT-PCR(Figs. 1A and 4A). Although *APOBEC3A* is a well-known cellular DNA cytidine deaminase that provides intrinsic immunity against viral infections, its expression could be enhanced by LPS exposure, leading to defense against invaded bacterial pathogens (Delviks-Frankenberry, Desimmie & Pathak, 2020; Mehta et al., 2012). SLC39A10 is a Zn transporter that participates in immune regulation by modulating intracellular Zn homeostasis (Hojyo et al., 2014; Miyai et al., 2014), and its expression is down-regulated following LPS stimulation and has been linked to immune responses (Gao et al., 2017). Conversely, we also found that both *MT2A* (metallothionein 2A) and *MT1E* (metallothionein 1E), which are involved in Zn absorption, were up-regulated by LPS treatment (Figs. 1A and 4A), which is in accordance with the results reported by Kim et al. (2022) and Wolf et al. (2020). These results implied that LPS might affect Zn transporter and metallothionein isoform mediated zinc influx in intestinal epithelial cells, which further influences immune responses.

In addition, KEGG pathway analysis revealed that the DEGs were enriched in inflammation response regulation related pathways, such as the TGF-beta signaling pathway and Ras signaling pathway (Fig. 3A). *INHBE* (inhibin subunit beta E) is a member of the TGF superfamily that rapidly increases after LPS stimulation but declines with stimulation time extension and plays important roles in inflammation responses (Jones et al., 2007; Wu et al., 2012). *ARF6* (ADP ribosylation factor 6) belongs to the Ras superfamily that is activated by LPS exposure and could trigger an inflammatory response, but the activation decreased with the extension of exposure time (Davis et al., 2014; Li et al., 2009). Consistently, both *INHBE* and *ARF6* were reduced after LPS treatment for 24 h in the present study (Figs. 1A and 4A).

Seventeen DEGs were identified in Caco-2 cells exposed to poly(I:C), including 9 and 8 up and down regulated genes, respectively (Fig. 1B). In line with the results obtained from the LPS exposure experiment, GO annotation analysis showed that cellular anatomical entity, intracellular, cellular process, biological regulation, and binding were the predominant functional terms (Fig. 2B). These results implied that the intestinal cellular structure might be damaged by bacterial or viral pathogen associated stimuli during the processes of infection (Hooper, 2015; Sun et al., 2019), even though most of the DEGs were specific to the two different infection processes (Fig. S2).

Additionally, DEGs annotated to GO terms such as immune system process and response to stimulus were also activated. Among these, *IFIT2* (interferon induced protein with tetratricopeptide repeats 2), *RUNX2* (RUNX family transcription factor 2), and *PLXNC1* (plexin C1) were up-regulated by poly(I:C) treatment and further verified by qRT-PCR (Figs. 1B and 4B). *IFIT2* is an important member of the interferon stimulated gene family that plays a crucial role in the antiviral innate immune response through the inhibition of viral replication (Chai et al., 2021). *IFIT2* is not transcribed under basal conditions but is induced to a high level after poly(I:C) treatment (Pang et al., 2018). *RUNX2* is an evolutionarily conserved transcription factor that has been described as essential for osteogenesis in the embryonic context, but a recent study revealed the participation of *RUNX2* in the antiviral response by modulating long-term memory and the survival and exhaustion of mature T cells (Mevel et al., 2019; Olesin et al., 2018). Importantly, both *RUNX1* and *RUNX2* belong to the *RUNX* family, the increased mRNA level of the former in response to poly(I:C) treatment has been previously reported (Yao et al., 2014), while the increased mRNA expression of the latter after poly(I:C) exposure was first observed in the present study. *PLXNC1* encodes a member of the plexin family that participates in the diverse immunoinflammatory processes and may be related to interferon gamma response (Ni et al., 2021). However, the relationship between *PLXNC1* expression and poly(I:C) stimulation has not been determined before and was revealed for the first time in this study.

On the other hand, autophagy and mTOR signaling pathway, which are intimately associated with antiviral response modulation, were identified by KEGG pathway analysis (Fig. 3B). Although the primary functions of autophagy are to maintain energy homeostasis and nutrient balance during stressful conditions, its pivotal roles in defending against invading viruses *via* autophagy-related proteins have been demonstrated recently (Foerster et al., 2022). *GABARAP* (gamma-aminobutyric acid type A receptor-associated protein) is a type of autophagy-related protein that is essential for autophagosomal maturation, and suppression of *GABARAP* could result in a reduction of autophagosomes that serve as a platform for virus replication (Blanchard & Roingeard, 2015; Li et al., 2017). The mTOR signaling pathway is a central regulator of many cellular processes including cell metabolism, growth, and proliferation, but it is over-activated during viral infection, which could benefit viral replication (Wang et al., 2021). *LAMTOR3* (late endosomal/lysosomal adaptor and MAPK and MTOR activator 3) is part of regulator complex that is required for activation of mTOR signaling pathway (Nada et al., 2014). Consequently, down regulation of *LAMTOR3* expression may repress the replication of viruses. Here, we found that the expression of *GABARAP* and *LAMTOR3* were inhibited by poly(I:C) treatment (Fig. 4B), which may be explained by poly(I:C) being a strong antiviral immunostimulant (Hafner, Corthesy & Merkle, 2013).

There were several major limitations in this study. One weakness was the short and single treatment time. Both bacterial and viral infections are time-dependent, and the time-varying stimulation studies could imitate different infection phases that aid in characterizing the dynamic processes of enteric pathogen infections. The small sample size and single exposure dose were other shortcomings. Additional samples utilized in different treatment groups with multiple doses would improve the statistical power and be beneficial to identify more DEGs for elucidating the underlying molecular mechanisms.

## Conclusions

In conclusion, we identified 70 and 17 DEGs involved in the specific responses of Caco-2 cells to LPS and ploy(I:C) simulation, respectively. Importantly, several DEGs related to immune regulation, inflammatory responses, and viral replication were verified. Our findings provid a basis for comprehensive understanding of the mechanisms that intestinal epithelial cells employ to defend against invading pathogens, which would be beneficial for developing prevention and treatment strategies for gut bacterial and viral diseases of farm animals.

##  Supplemental Information

10.7717/peerj.15459/supp-1 Data S1 qPCR validation

10.7717/peerj.15459/supp-2 Table S2 Differentially expressed genes (DEGs) in Caco-2 cells under poly(I:C) stimulation

10.7717/peerj.15459/supp-3 Figure S1 PCA analysis of the gene expression profile of Caco-2 cells under LPS (A) and poly(I: C) (B) treatment

10.7717/peerj.15459/supp-4 Figure S2 The common and specific DEGs in Caco-2 cells exposed to LPS and poly(I: C)

10.7717/peerj.15459/supp-5 Table S1 Differentially expressed genes (DEGs) in Caco-2 cells under LPS stimulation

10.7717/peerj.15459/supp-6 Table S3 GO annotation of DEGs response to LPS and poly(I:C) exposure

10.7717/peerj.15459/supp-7 Table S4 KEGG enrichment analysis of DEGs response to LPS and poly(I:C) exposure

## Additional Information and Declarations

### Data Availability

The following information was supplied regarding data availability: The PCR data are available in the Supplemental Files. The transcriptome sequencing data is available at the China National GeneBank DataBase (CNGBdb): CNP0003540 (CNS0619566, CNS0619567, CNS0619568, CNS0619569, CNS0619570, CNS0619571, CNS0619572, CNS0619573, CNS0619574, CNS0619575, CNS0619576, CNS0619577).
