# Supplementary figures and images for: Alterations in gene expressions of Caco-2 cell responses to LPS and ploy(I:C) stimulation
Source: PeerJ. 2023 Jun 7;11:e15459. doi: 10.7717/peerj.15459 (PMC10257391; doi:10.7717/peerj.15459)

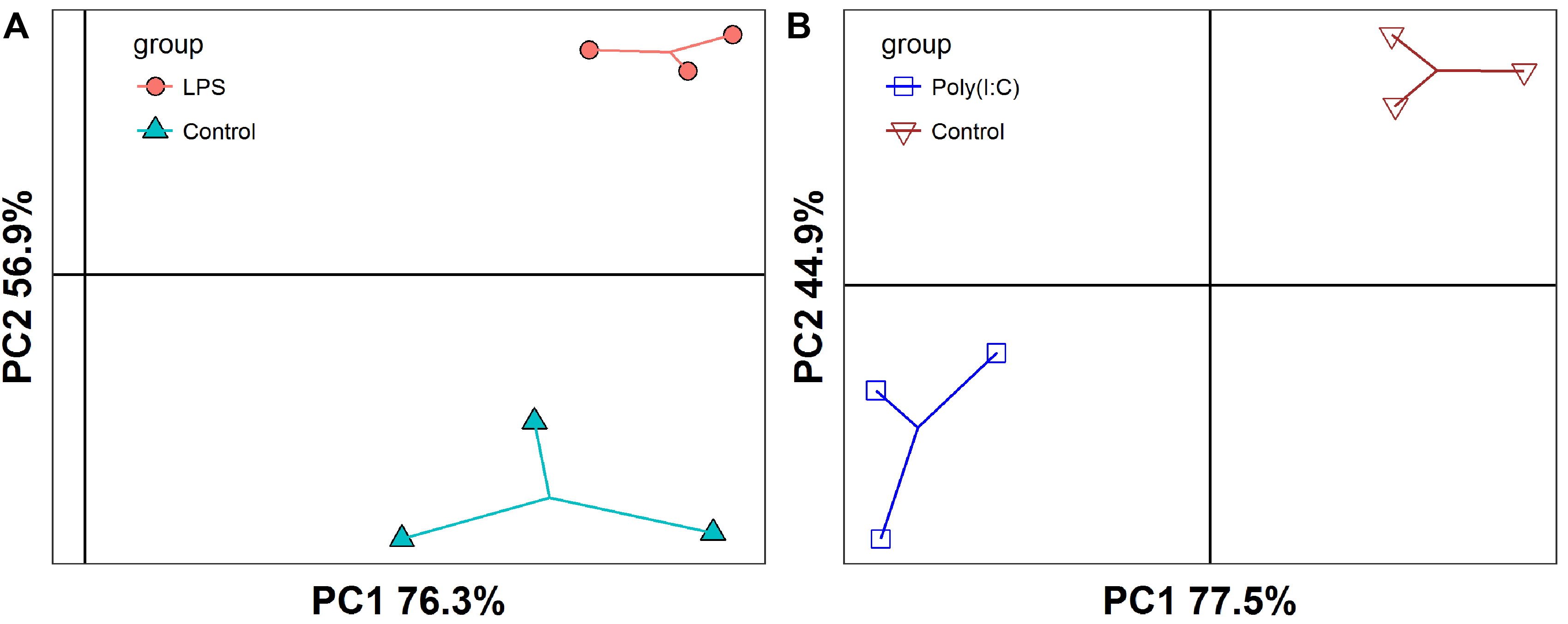

Supplement: Figure S1 [file peerj-11-15459-s003.png]

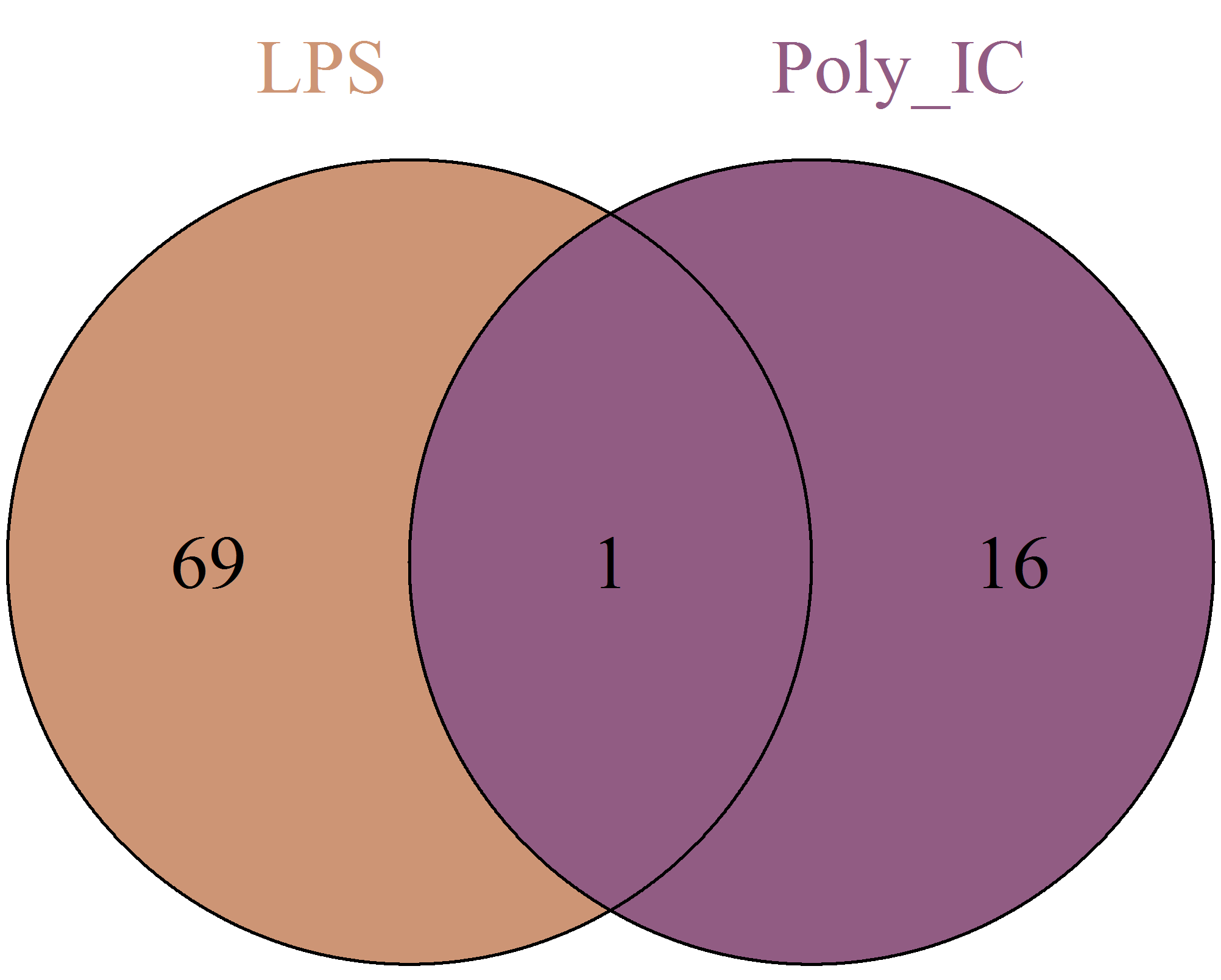

Supplement: Figure S2 [file peerj-11-15459-s004.png]
